# Could Modifying the Bagolini Glasses Improve the Reliability of Responses?

**DOI:** 10.22599/bioj.139

**Published:** 2019-12-09

**Authors:** Anna O’Connor, Laurence Tidbury

**Affiliations:** 1 University of Liverpool, GB

**Keywords:** binocular vision, Bagolini glasses, suppression, orthoptics, sensory fusion

## Abstract

**Aims:**

Bagolini striated lenses are a useful test of binocular vision, but the variations in the striations (frequency and thickness) can impact on the perceived image. Also, responses can be difficult to interpret in young children. Therefore, the aims of this project were to evaluate the impact of striation frequency and the addition of coloured filters on subjective responses.

**Methods:**

Three sets of striated lenses were made (small, medium and large striations), each produced in two forms (both lenses clear, or with a red and blue lens). Also, Bagolini glasses (Optiker Ryser) were used, with and without the addition of red and blue filters. Subjects were asked to report what they perceived, with subsequent questions regarding the number and length of lines.

**Results:**

Forty-two adult subjects were tested, with uniocular VA ranging from –0.18 to 1.10 logMAR (mean 0.08 ± 0.25). The number of lines seen when varying the line thickness did not vary between coloured and clear lenses (post-hoc analysis following ANOVA, p > 0.1 in all comparisons). Adding red/blue filters to the original Bagolini glasses did not alter the rates of subjects perceiving a cross (Chi-square, p = 0.8). However, the laser-cut lenses produced a significantly shorter light streak than the original lenses (One-way ANOVA, p < 0.001), but the colour of the filters made no difference to the length of streak perceived (Tukey’s Test, p = 0.20).

**Conclusions:**

The addition of coloured filters did not impact on the responses given to the original or laser-cut lenses, suggesting this modification may aid responses in children. However, further evaluation is required with finer striations and thinner lenses to improve the visibility of the lines.

## Introduction

The assessment of the status of binocular vision (BV) is a fundamental component of the orthoptic investigation, which includes the identification of the presence or absence of BV. There are a number of ways of determining this, with the choice being determined by patient characteristics or clinician preference. One such test is the Bagolini striated lenses, which are a commonly used test to determine the presence of sensory fusion, created 40 years ago (Bagolini 1976; Bagolini 1999). These lenses have been used in a range of studies of amblyopia (Li et al. 2013), strabismus (Dickmann et al. 2013), motor fusion (Schultinga et al. 2013) and neurological lesions (Hirai et al. 2005), demonstrating their relevance in clinical orthoptics.

This simple, quick and easy-to-administer test can provide important information about the status of BV in a range of patients, for example in young children with strabismus where the presence (or lack thereof) of sensory fusion impacts on management choices. For example, the presence of sensory fusion influences choices in relation to strabismus surgery and whether the aim is to align the eyes, over- or under-correct (Ansons and Davis 2014). However, it requires a level of cognitive ability to ensure an accurate response. In addition, based on observation the striations on the current Bagolini glasses not only differ between each set, but also vary in length, frequency, thickness and continuity. As a result, multiple lines may be seen, in addition to the intended image, with the potential to create difficulty in the interpretation of responses. A newer pair of Bagolini glasses has potentially addressed some of these issues with less distortion, purely by virtue of being new, with no damage reducing light transmission and introducing unintended distortions; however, the variation in the frequency and thickness of the lines remains.

To try to reduce the variability in the image created, a laser cutter was used to generate the striations. As a result, the lines perceived by the subject may be more consistent between different lenses, seeing a clearer single line by each eye, and therefore improve the reliability of responses. However, it is not known whether a particular frequency of stripes will create a clearer image. In addition, the use of coloured lenses may provide an extra detail for a patient to describe, enabling easier interpretation of the response from each eye. Therefore, the aims of this project were to:

1. Evaluate different striation frequencies and their impact on subjective response.
2. Evaluate the addition of a red and blue filter on subjective response.

## Methods

### Producing the lenses

A laser cutter with pre-set settings was used to create three sets of striated lenses from acrylic with the same diameter as the Bagolini glasses but a frequency of 39 striations per 3 cm (small), 7 striations per 3 cm (medium) and 4 striations per 3 cm (large). The striations were continuous and evenly spaced. Each set of lenses was produced using a clear lens and a red and blue pair. In addition, the original plastic Bagolini glasses (Optiker Ryser) were also used (approximately 30 striations per 3 cm), with and without the addition of red and blue filters, simply taped to the glasses at the edges. This resulted in a total of eight pairs of lenses. All coloured lenses were cut from a single sheet of plastic for consistency.

### Subjects

Subjects were recruited from the University of Liverpool, informed consent was obtained prior to assessment from all subjects. The research protocol observed the tenets of the Declaration of Helsinki and was approved by the University of Liverpool ethics committee. Subjects were aged 18 years or older; the only exclusion criteria was prior knowledge of the test and possible responses (determined by asking the subject and excluding any staff or students from orthoptics), to eliminate any potential bias in the responses. This was a Nuffield research summer student project, providing two year-12 students with the opportunity to participate in a research project and train in the test procedures required. As the testing was undertaken by non-orthoptic students, no testing on eye alignment was possible, but the aim was to determine any differences between lenses, irrespective of eye alignment.

All testing was conducted with the subjects wearing their habitual correction. Prior to testing with the striated lenses uniocular visual acuity (VA) was assessed with the ETDRS chart.

### Test procedure

All testing was conducted in a dark room, to ensure that responses were based on a single light source. An ophthalmoscope (Keeler vista 20) was used as the light source (largest size light used), fully charged between each subject to ensure stability of temperature and intensity. To maintain a consistent test distance, a chin rest was used, with the light source fixed in place at 33cm. Test order was counterbalanced across subjects.

For each set of lenses, subjects were tested under binocular conditions and asked the following questions (no other explanation provided so that subjects responses were not influenced):

- How many lines can you see going through the light source?
- What is the overall shape that is made by the lines?
- How long are the brightest lines, on a scale of 1–10 with 1 being the smallest possible line and 10 being to the edge of your vision?

## Results

A total of 42 subjects were tested, with uniocular VA ranging from –0.18 to 1.10 logMAR (mean 0.08 ± 0.25), seven of whom had an interocular acuity difference (mean 0.17 ± 0.27) of more than 0.2 logMAR.

### Impact of width of striations on perceived response

**Figure 1:**
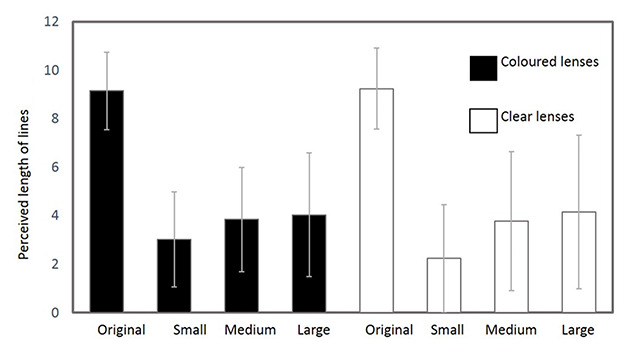
A bar chart of the mean length of line perceived with each set of lenses, error bars showing the standard deviation.

The longest lines were perceived using the original glasses (Figure 1), with and without coloured lenses. A one-way ANOVA demonstrated a statistically significant difference in the length of line perceived between the lenses based on striation frequency (p < 0.001). Post hoc comparisons (Tukey’s Test) showed the only significant differences were between the original and any laser-cut lenses, with a longer line perceived using the Bagolini lenses. There was no statistically significant difference based on lens colour (p = 0.20).

### Impact of coloured lenses on the perception of a cross response

Responses of the shape seen included cross hatch, triangle, line and mesh. Given the variability and the fact that the only response indicating binocular vision is cross, responses are grouped into cross or other. Comparison of the original lenses with/without the coloured lenses showed no statistically significant difference (Chi-square, p = 0.8) Table 1.

**Table 1:** Rates of responses of the perception of a cross through all lenses.

	Coloured lenses	Clear lenses

**Cross**	23	24
**Other response**	19	18

### Impact of coloured lenses on the number of lines perceived

**Figure 2:**
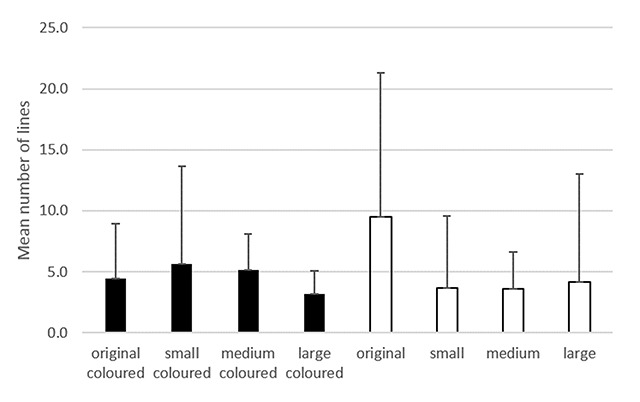
Bar chart showing the mean number of lines perceived through each set of lenses, with error bars showing the standard deviation.

As shown in Figure 2, the mean number of lines perceived ranged from three to nine. Analysis of variance demonstrated a statistically significant difference in the number of lines produced by the different lenses (p = 0.02). Post-hoc analysis showed no statistically significant difference between the coloured lenses and the clear lenses (p > 0.1 in all comparisons of equal striation thickness). However, the original glasses produced a statistically significant difference, with more lines, when compared to all the clear lenses (p < 0.03 in all cases).

**Figure 3:**
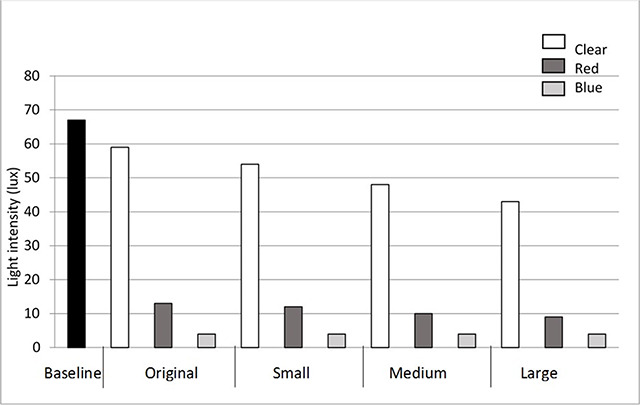
The light intensity transmitted through each lens.

During testing, responses from subjects indicated that the coloured lenses impacted on the transmission of light, resulting in the light source being perceived as less bright. Therefore further testing was undertaken to measure the light intensity through each lens using a light meter. As shown in Figure 3, there is a reduction in the light intensity measured through the coloured lenses.

## Discussion

The results of this study demonstrated that when using the laser-cut lenses colour did not impact the response. In addition, fewer lines were perceived than for the original Bagolini glasses, but the length of line perceived was shorter. This reduction in the number of lines combined with coloured lenses suggests a potential benefit of coloured laser-cut lenses.

In relation to the applicability of this test to clinical practice, this first phase of evaluation has some limitations in that over half of the subjects in this opportunistic sample reported a sensory fusion response, and testing conditions were not reflective of standard clinical practice. The consistency in the testing conditions does facilitate direct comparisons between responses to the different lenses, the limited number of subjects with suppression or intermittent strabismus means it is not known whether the coloured lenses have a greater impact on dissociation. Bagolini glasses are said to be ‘minimally dissociating’ (Ansons & Davis 2014) and that it is rare to produce a diplopic response from dissociation due to the glasses (Bagolini 1999), due to the limited impact of the striated lenses. However, no data were found to support these statements, so the impact of coloured lenses may be minimal.

While the aim was to produce a simpler test with easy-to-interpret responses, the reduction in number of lines is a positive finding, but the shorter lines seen through the laser-cut lenses may make the response less obvious to the patient. This response may be attributable to the striation frequency of the Bagolini glasses being higher than the laser-cut lenses, with much finer striations. The striation widths were also much narrower than those produced for this study. For this study, pre-set settings were used but with careful calibration, the laser beam could be focused to 0.1mm to provide thinner striations, which should result in longer light streaks being apparent.

One finding which significantly impacted the image seen was the reduced light transmission through the coloured lenses. The darker lenses were used so that the image seen would be a clear definite colour; however, in future designs thinner plastic will be used to improve light transmission, while maintaining a clear coloured response that the patient can report. Figure 3 suggests that the red light would be more prominent than the blue, but this measurement would be influenced by the spectrum of the light source; for example, halogen lights contain a greater amount of red light. The addition of colour does provide an extra descriptor for children who may not provide a clear response in terms of the shape seen, but may be able to report on whether a specific colour is present or absent.

No clinical information was collected regarding the subjects’ eye alignment as this was a student project run by non-orthoptic students. While this limits the interpretation of the findings, the purpose of the study was to make comparisons, which was achievable without additional clinical data. Further testing will include a full orthoptic assessment.

In conclusion, the addition of blue and red filters did not impact the responses given, suggesting this modification may be beneficial to clinical testing as it provides respondents with another visual cue to what is seen. However, further modifications are required to improve the visibility of the lines seen, such as using thinner striations at a higher frequency and potentially reduce the number of lines seen.
